# The feasibility of an exercise intervention after robotic-assisted radical cystectomy for urinary bladder cancer, prior to the CanMoRe trial

**DOI:** 10.1186/s40814-024-01443-1

**Published:** 2024-01-22

**Authors:** Andrea Porserud, Patrik Karlsson, Malin Nygren-Bonnier, Markus Aly, Maria Hagströmer

**Affiliations:** 1 Division of Physiotherapy, Department of Neurobiology, Care Sciences and Society, Karolinska Institutet, Alfred Nobels Allé 23, 23100, 141 83 Huddinge, Sweden; 2 Medical Unit Occupational Therapy and Physiotherapy, Women’s Health and Allied Health Professionals Theme, Karolinska University Hospital, 171 76 Stockholm, Sweden; 3 Department of Molecular Medicine and Surgery, Karolinska Institutet, 171 77 Stockholm, Sweden; 4 Patient Area Pelvic Cancer, Cancer Theme, Karolinska University Hospital, 171 76 Stockholm, Sweden; 5 Academic Primary Health Care Centre, 113 65 Stockholm, Region Stockholm Sweden

**Keywords:** Complex intervention, Physical activity, Physiotherapy, Postoperative complications, Primary health care

## Abstract

**Background:**

Complications after radical cystectomy for urinary bladder cancer are common. Physical activity after surgery is thought to reduce complications. However, patients with urinary bladder cancer have low levels of physical activity, and interventions supporting physical exercise are needed. This study aimed to evaluate the feasibility of a physical exercise intervention in primary health care. One of the aims of the larger clinical trial will be to reduce complications.

**Methods:**

Patients with urinary bladder cancer and who were scheduled for a robotic-assisted radical cystectomy were recruited from Karolinska University Hospital, between February and May 2019. The patients had to be mobile, understand Swedish, and live in Stockholm. The exercise programme was conducted at one primary health care setting over 12 weeks. The exercise programme included supervised aerobic and strengthening exercises, which were performed twice a week, as well as daily walks. Feasibility was measured with process feasibility, including eligibility criteria, adherence, and acceptability, and scientific feasibility, including the ability of outcomes to indicate change, safety, and progression in the exercise programme.

**Results:**

Ten patients with a median age of 70 years (min 53–max 86) were included. Adherence to all parts of the intervention was not feasible because of patients’ postoperative complications, resulting in dropouts. For the patients who took part in the exercise programme, adherence and acceptability for the exercise period were feasible, but the 6-min walk test was not feasible at discharge from the hospital. Physiotherapists in the primary health care setting perceived the process as feasible. Moreover, the ability of outcomes to indicate change and progression in the exercise programme was feasible, meanwhile no adverse events were registered.

**Conclusions:**

The exercise intervention was feasible for the patients that took part in the exercise programme, with respect to safety and progression through the exercise programme. Furthermore, this study suggests that some improvements needed to be implemented in the process, prior to the upcoming randomised controlled trial.

## Key messages regarding feasibility

- What uncertainties existed regarding the feasibility?
  There is limited information on the effects of exercise after radical cystectomy for urinary bladder cancer. This study primarily investigated the feasibility regarding adherence to an exercise programme, acceptability of the exercise period and physical tests, safety for the patients, and the possibility of progression in the exercise programme, prior to a randomised controlled trial.
- What are the key feasibility findings?
  Several patients were affected by postoperative complications, which resulted in dropouts. For the patients that remained in the study, adherence to the exercise programme and acceptability for the exercise period were feasible, but the 6-min walk test was not feasible at discharge tests. No adverse events occurred; therefore, safety was feasible. Progression in the exercise programme was also deemed to be feasible.
- What are the implications of the feasibility findings for the design of the main study?
  To account for dropouts, more patients will be added after the power calculation. At discharge, a gait speed test will be added to measure physical function, which is believed to be easier for the patients to perform. Also, instructions to patients regarding the 6-min walk test will be revised.

## Background

Mobilisation at the hospital ward followed by physical activity at home is thought to reduce complications after abdominal surgery [1]. Surgery is the most common treatment for solid cancer tumours and is often combined with either chemotherapy or radiotherapy, or both [2]. Patients who undergo radical cystectomy (RC) for urinary bladder cancer (UBC) are frail, have a high degree of comorbidity, and have a mean age of 70 years; all of which are associated with postoperative complications [3–5]. After robotic-assisted radical cystectomy (RARC) in the treatment of UBC, it is estimated that between 19 and 75% of patients need to be readmitted to the hospital due to complications occurring within 30- or 90-day periods [6]. The most frequent complications after RC are venous thrombosis, pulmonary complications, infections, ileus, postoperative anaemia, and metabolic acidosis [7–10]. In recent studies, the 5-year recurrence-free survival rates have been reported to vary between 58% [11] and 70% [12].

Physical activity is associated with improved health-related quality of life (HRQoL) in UBC survivors, and functional performance after RC due to UBC has been shown to correlate with overall survival rates [13, 14]. The physical activity guidelines for patients with cancer suggest doing at least 150 min of moderate-intensity physical activity per week and muscle-strengthening exercises twice a week [15]. Patients with UBC, similar to patients with other types of cancer, have shown difficulties in achieving the physical activity guidelines [16, 17]. Nevertheless, studies have shown that physical activity and exercise are associated with decreased UBC risk [18]. Moreover, physical exercise interventions after RC are scarce. One randomised controlled trial (RCT) reported that enhanced mobilisation and early physical exercise after RC positively affected several domains of HRQoL [19]. We also tested a model for physical rehabilitation after RC, which consisted of 12 weeks of exercise twice a week at the hospital, after hospital discharge. The model showed both short- and long-term effects on physical function and HRQoL, but the patients had difficulties to attend the exercise due to long travel distances [20].

To find optimal ways of supporting these patients with rehabilitation is crucial. Therefore, an RCT was designed, called the CanMoRe trial. The main aim of this trial will be to evaluate the impact of an exercise intervention in primary health care (PHC) for patients undergoing RARC for UBC [21]. However, due to the complexity of the intervention—which includes both the hospital setting and several PHC settings, along with different categories of healthcare professionals— there was a need to test feasibility prior to the planned RCT [22, 23]. Hence, the aim of this study was to evaluate the process feasibility and the scientific feasibility of an exercise intervention for patients who have undergone RARC due to UBC.

## Methods

### Study design

A prospective one-group pre-test/post-test design was used to evaluate the feasibility of an exercise intervention. The study was approved by the regional ethical review board in Stockholm (Dnr 2012/2214–31/4) and the Swedish Ethical Review Authority (Dnr 2020–01356). The reporting is guided by the CONSORT extension to pilot and feasibility trials [24].

### Participants and settings

Patients with UBC who were scheduled for a RARC at Karolinska University Hospital between January 02, 2019, and March 05, 2019, were asked to participate in the study. According to the inclusion criteria, patients had to be mobile with or without a walking aid and be able to speak and understand Swedish without an interpreter. Patients had to live in Region Stockholm and live approximately within 30 min to the chosen PHC in Stockholm, where the exercise took place. Patients who were scheduled for palliative surgery were not included in the study.

### Procedure

Screening of eligible patients in medical records was conducted by two researchers, both of whom were physiotherapists. Patients were asked to participate in the study during a preoperative meeting with a registered nurse, approximately 1 week prior to the scheduled surgery. Patients received written information about the study, and the researcher provided more information and asked about participation via a telephone call. Written informed consent was obtained before surgery. After patients agreed to participate, the researcher informed physiotherapists at PHC. When patients were discharged from the hospital the researcher sent a referral to PHC, alerting physiotherapists of the need to contact the patient to begin exercise in the third week following discharge. Three physiotherapists at PHC received written and verbal information and education about RARC, restrictions, and the specified exercise programme. As is standard in this type of care, patients paid for their visits at the PHC.

#### Measurements

Measurements that are planned to be used in the RCT were used in this feasibility study to evaluate their process and scientific feasibility [21]. The patients performed physical tests and filled out questionnaires on the day before surgery, at discharge from the hospital, 4 months after surgery, and the researcher conducted the measurements at the hospital. Physical function was measured with the 6-min walk test where the patients were asked to walk as far as possible for 6 min [25, 26]. Grip strength was measured with Jamar [27], leg strength via a 30-s chair stand test [28], HRQoL with EORTC QLQ C-30 with the addition of the EORTC BLM-30 [29, 30], fatigue with the Piper Fatigue Scale [31], psychological well-being with the Hospital Anxiety and Depression Scale (HADS) [32], pain with the Numeric Rating Scale (NRS) [33], and habitual physical activity was measured with the activity monitor ActivPAL [34, 35]. Habitual physical activity was measured for seven consecutive days after discharge from the hospital and for seven days after the 12-week exercise period. Also, data regarding the patients’ preoperative demographic and characteristics, surgical procedure, and postoperative characteristics were retrieved from the patient’s medical records.

### Intervention

Patients received standard care regarding preoperative information about early mobilisation and individualised physiotherapy following surgery. During discharge from the hospital, the patients also received standard physiotherapeutic information about the importance of physical activity and avoiding lifting heavy objects. Patients were contacted by PHC physiotherapists at home to schedule the first exercise session.

The exercise programme was based on international recommendations for patients with cancer, but each programme was individualised with respect to pain or postoperative feebleness. The exercise programme duration was 12 weeks, twice a week, and consisted of aerobic exercise (30 min/session) and strengthening exercises. The exercise programme also included exercises for abdominal muscles, including pelvic floor exercises, to reduce the risk of stoma hernia [36]. However, restrictions on abdominal muscle exercises were followed until 6 weeks post-surgery. The exercise programme was consequently divided into two blocks, with less load on abdominal muscles during the first 6 weeks after surgery. The aerobic exercise was performed in intervals, at moderate intensity (Borg’s Rating of Perceived Exertion scale, RPE-scale 12–13) for the first 5 weeks, and thereafter at moderate to high intensity (Borg’s RPE-scale 12–15). Five strengthening exercises were prescribed: two for the lower body and three for the upper body, comprising endurance strength, at 50–70% of 1 repetition maximum (RM) (2 × 15 repetitions) the first 2 weeks and at 65–75% of 1 RM (2 × 10 repetitions) from the third week. Patients also received recommendations to take daily walks and to set goals for daily steps, together with the physiotherapists. Physiotherapists regularly supported the patients’ daily walks by using individual goal-setting, feedback, and self-monitoring. The patients used a pedometer or mobile phone for self-monitoring of daily step counts. Once a week the physiotherapist gave oral feedback on the steps taken, and new individual goals for daily steps were set. Progression of aerobic and strengthening exercises and daily walks were documented by physiotherapists in exercise protocols. As is standard, PHC physiotherapists recommended continued physical activity and exercise after the 12-week exercise period.

### Feasibility outcomes

Evaluation of the feasibility objectives was guided by the recommendations by Thabane et al. with a focus on process feasibility and scientific feasibility [37]. Process feasibility assesses the feasibility of the processes that are the key to the success of the main study, and scientific feasibility assesses treatment safety, dose, response, effect, and variance of effect [37]. Definitions of feasibility outcomes, assessment methods, and feasibility thresholds are presented in Table 1.

**Table 1:** Feasibility outcomes

Type of outcome	Definition	Assessment method	Feasibility threshold
**Process feasibility**			
Eligibility	Adequate eligibility criteria	N of inadequate inclusions	0 events
Adherence * To all parts*^a^* of the intervention*	N of patients who attended	Study protocols Exercise protocols	≥ 50%
**For patients remaining in the study**			
Adherence			
* To the exercise programme*	N of exercise sessions attended out of those planned	Exercise protocols	≥ 50%
* To begin the exercise programme*	N of patients that started exercise the latest within the third week, after hospital discharge	Medical records Exercise protocols	≥ 50%
Acceptability			
* Exercise period*	N that withdrew or dropped out after starting the exercise period	Exercise protocols Medical records	< 50%
* Physical tests* * Questionnaires*	N of tests/questionnaires performed out of those planned	Study protocols	≥ 80% of each test/questionnaire
* Activity monitoring*	N of patients that used the activity monitor and N of valid days	Activity monitor logs	≥ 50% of patients use ≥ 4/7 valid days
**Scientific feasibility**			
Ability to indicate a change * Physical tests* * Questionnaires* * Activity monitoring*	Measurements must vary between test occasions, for each patient	Study protocols Activity monitor logs	> 0% variation
Safety * Exercise programme* * Physical tests*	N of adverse events	Exercise protocols Study protocols	0 events
Possibility of progression in the exercise programme	N of patients who proceeded from block one to block two	Exercise protocols	≥ 80%

^a^All parts = tests preoperatively, and at discharge, physical exercise programme, tests at 4 months follow-up. *N* = numbers

#### Process feasibility

Process feasibility was measured with eligibility criteria, adherence, acceptability of the exercise period, physical tests, questionnaires, and activity monitoring and acceptability by PHC physiotherapists.

*Eligibility criteria* were defined to be inadequate if patients were invited to participate that should not have been invited, which was clear when researchers met the patient face to face the day before surgery.

*Adherence* to all parts of the intervention, to the exercise programme, and to when the patients started the programme were evaluated using patients’ medical records, study protocols, and exercise protocols, which PHC physiotherapists filled out.

*Acceptability of the exercise period* was assessed through measuring patient dropouts, pauses during the exercise period, and reasons for withdrawal or pauses. These were evaluated through exercise protocols and patients’ medical records.

*Acceptability of physical tests, questionnaires, and activity monitoring* was evaluated by study protocols and activity monitor logs.

*Acceptability by physiotherapists at PHC* was measured through hospital discharge referrals and their opinions on the exercise programme and administration. These were evaluated via a study-specific free-response questionnaire, which included 12 questions, answered by physiotherapists at the end of the study.

#### Scientific feasibility

Scientific feasibility was evaluated through the ability of the physical tests, questionnaires, activity monitoring to indicate change, the safety of the exercise programme and physical tests, and the possibility of progression in the exercise programme.

*Ability of physical tests, questionnaires, and activity monitoring to indicate change* was evaluated since the measurements seldom have been used for patients often affected by postoperative complications. Both the surgery and complications could result in the absence of variation between tests at discharge and at four months.

*Safety of the exercise programme and physical tests* were evaluated with exercise protocols or through researchers conducting the tests.

*The possibility of progression in the exercise programme* was defined as the possibility for patients to proceed from block one to block two, increased weight used for the strengthening exercises, performing aerobic interval exercise, and increased intensity for aerobic exercise, as registered in exercise protocols.

### Statistical analysis

Descriptive statistics were used to present the demographic and clinical characteristics of the patients, as well as the results of physical tests, activity monitor use, and the questionnaires’ ability to indicate change. IBM SPSS statistics version 27 was used for the statistical analyses.

## Results

**Fig. 1:**
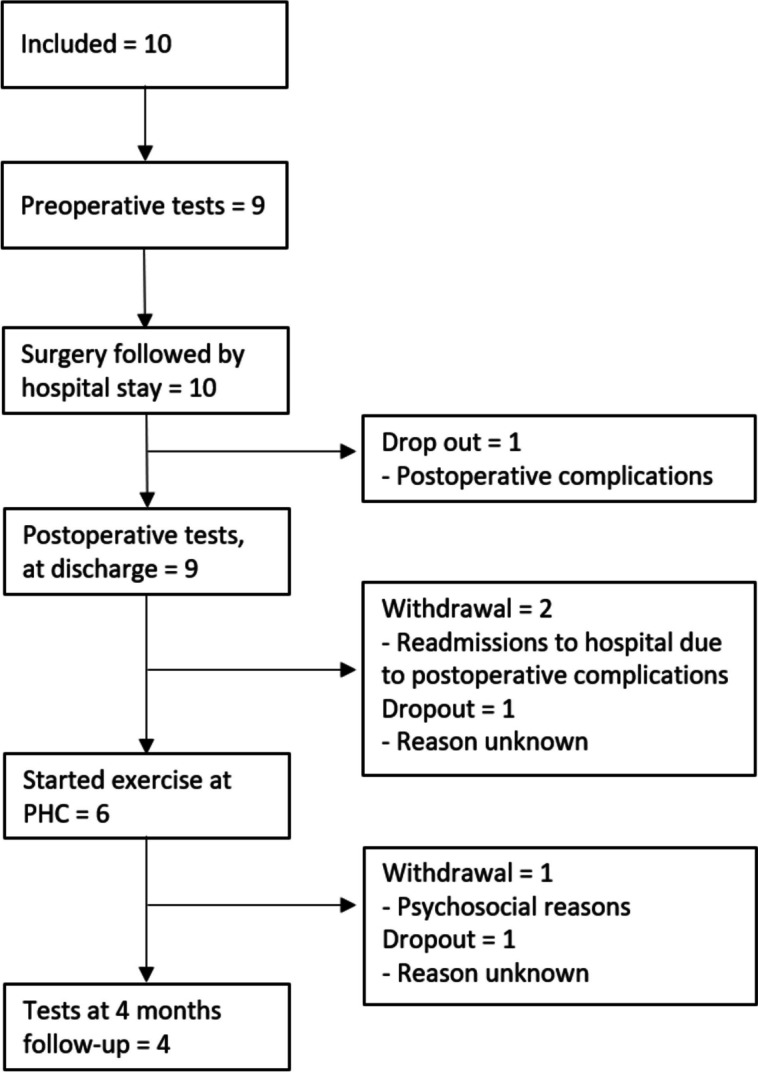
Flow chart of the study

Ten patients: eight men and two women were included in this study (Fig. 1). Of these ten patients, two withdrew from participation and two dropped out before starting the exercise programme at PHC (Fig. 1). Also, one patient withdrew from participation and one patient dropped out before the follow-up tests 4 months after surgery. The ten patients’ median age was 70 years (min 53–max 86) and the median age of the four patients who performed the follow-up tests was 77.5 years (min 70–max 86). Further demographic and clinical characteristics are presented in Table 2.

**Table 2:** Preoperative demographic and clinical characteristics of study participants (*n* = 10)

Body mass index, kg/m^2a^	23.9 (19.1–26.6)
Smoking status, *n*	
*Never smoked*	2
ASA-class, *n*	
*2*	8
*3*	2
Tumour grade, *n*	
*TaG3 CIS*	1
*T2G2*	1
*T2G3*	4
*T2G3 CIS*	4
Neoadjuvant chemotherapy, *n*	6
**Surgery**	
Urinary diversion	
* Ileal conduit, n*	4
* Orthotopic neobladder,* n	6
Operative time, minutes^a^ (*n* = 8)	293 (270 – 430)
**Postoperative**	
Time to first stool, number of postoperative days^a^	3 (2 – 10)
In need of a higher level of care, *n*	2
Length of stay, (days at hospital), *n*^a^	7 (5 – 15)
**Discharge**	
Discharged to inpatient rehabilitation clinic, *n*	5
Readmission to hospital within 30 days after discharge, *n*	5
Readmission to hospital within 90 days after discharge, *n* (*n* = 8)	2

*Abbreviations*: *ASA* American association of Anesthesiologists, 1 = low comorbidity, 4 = high comorbidity, *CIS* = Carcinoma in situ

^a^Presented as median (min–max)

### Feasibility outcome results

#### Process feasibility

##### Eligibility criteria

The eligibility criteria were not feasible since they were not specific enough for one patient, and this patient was not included in the study. When the researcher met the patient the day before surgery, it was clear that a cognitive impairment existed that had not been acknowledged. The patient did not understand the study information and did not want to participate in the study.

##### Adherence

Four (40%) patients attended all parts of the intervention, and consequently, the intervention was not feasible. However, adherence to the exercise programme was feasible. The median number of sessions attended was 12 (min 2–max 22) out of a maximum of 24 planned sessions, for the six patients that started the exercise programme. Three of the six patients started the exercise at PHC within the third week after discharge from the hospital, deemed as feasible.

##### Acceptability of the exercise period

Two out of six patients withdrew or dropped out, before the follow-up tests, which was defined as feasible. The same two patients experienced pauses during their exercise periods due to medical reasons, but they were not readmitted to the hospital during the exercise period. Three of the six patients started the exercise programme but did not exercise through all 12 weeks, the reasons for which are unknown.

##### Acceptability of physical tests, questionnaires, and activity monitoring

The number of physical tests and questionnaires performed out of the number that were planned are presented in Table 3. The 6-min walk test was not found to be feasible to conduct at discharge from the hospital. Also, one patient did not perform the preoperative physical tests and questionnaires due to a stressful time schedule. Another patient did not perform the postoperative physical tests and questionnaires due to complications which resulted in that patient dropping out of the study. Seven out of nine patients used the activity monitor after hospital discharge, and the median number of valid days of monitor use was 7 (min 4–max 7). At four months, three out of four patients used the activity monitor, and the median number of valid days of monitor use was 3 (min 1–max 7). Valid days at four-month measurements were thereby not feasible.

**Table 3:** Physical tests and questionnaires that were performed out of numbers planned, for patients remaining in the study on different occasions

	**Preoperative**	**At discharge**	**4-month follow-up**
Six-minute walk test^a^	9/10	4/9	4/4
Hand grip strength with Jamar, right hand^b^	9/10	9/9	4/4
Hand grip strength with Jamar, left hand^b^	8/10	9/9	4/4
30-s chair stand test^c^	9/10	8/9	4/4
Questionnaires	9/10	9/9	4/4

^a^Measured in meters, ^b^Measured in kilograms, ^c^Measured as in number of sit to stand transitions

##### Acceptability by physiotherapists

PHC physiotherapists were satisfied with the hospital referrals, but they expressed those patients who lived further away from PHC had some difficulties with travel distance. It was considered feasible to begin the exercise period the third week after hospital discharge, except for patients who suffered from postoperative complications early after discharge. Regarding the exercise programme, it was possible to achieve progression in strengthening exercises. A few patients expressed some discomfort in the surgical area when they exercised with too much flexion in the hips, but according to the physiotherapists, it was easy to adjust the exercise. Setting goals for daily steps together with patients was also acceptable to the physiotherapists. Overall, patients were perceived as highly motivated and participated actively in the exercise programme.

#### Scientific feasibility

##### Ability of the physical tests, questionnaires, and activity monitoring to indicate change

**Fig. 2:**
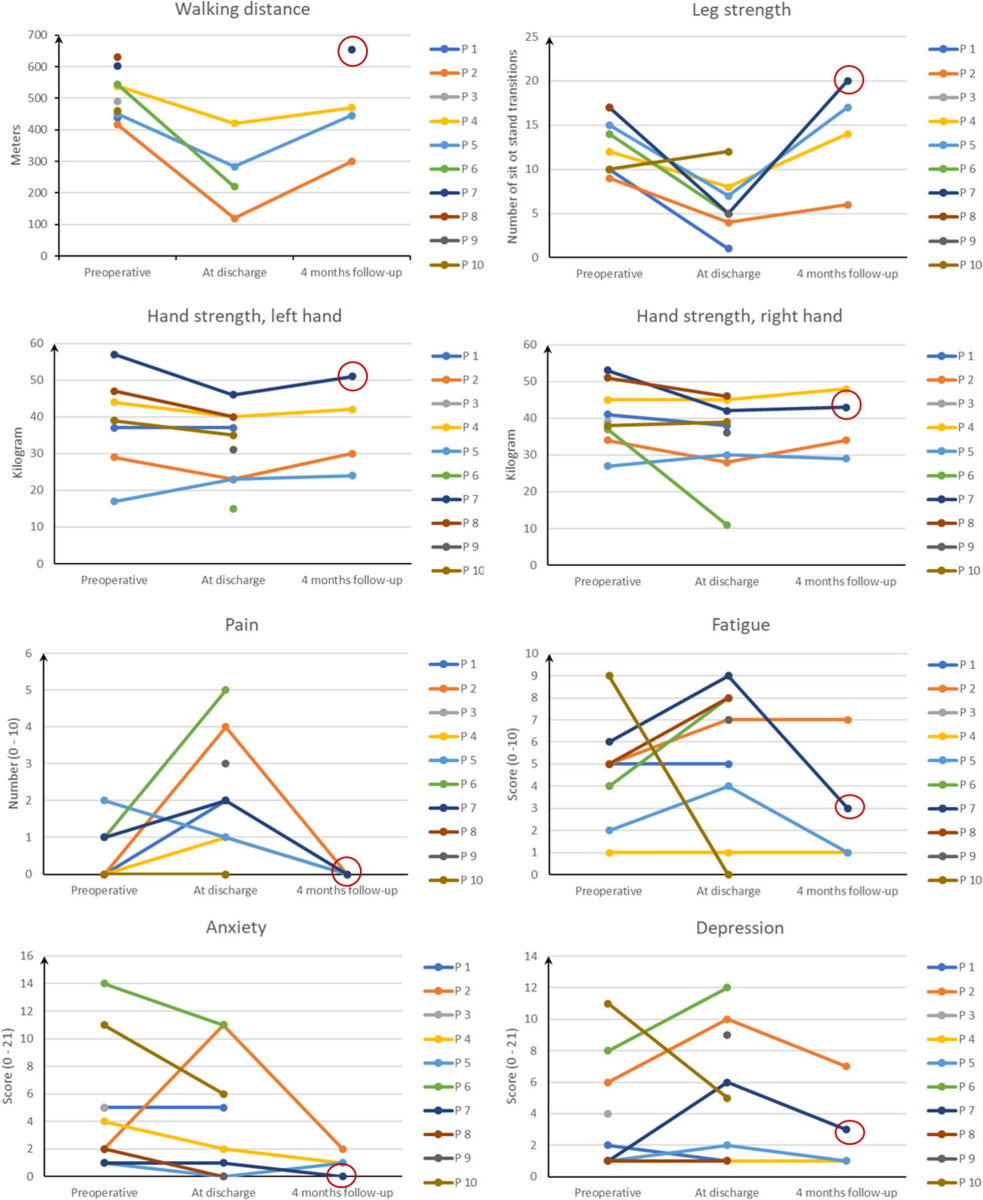
Descriptive data from physical tests and questionnaires. The patient who exercised 22 out of 24 sessions has been highlighted with a circle, at 4 months follow-up

**Fig. 3:**
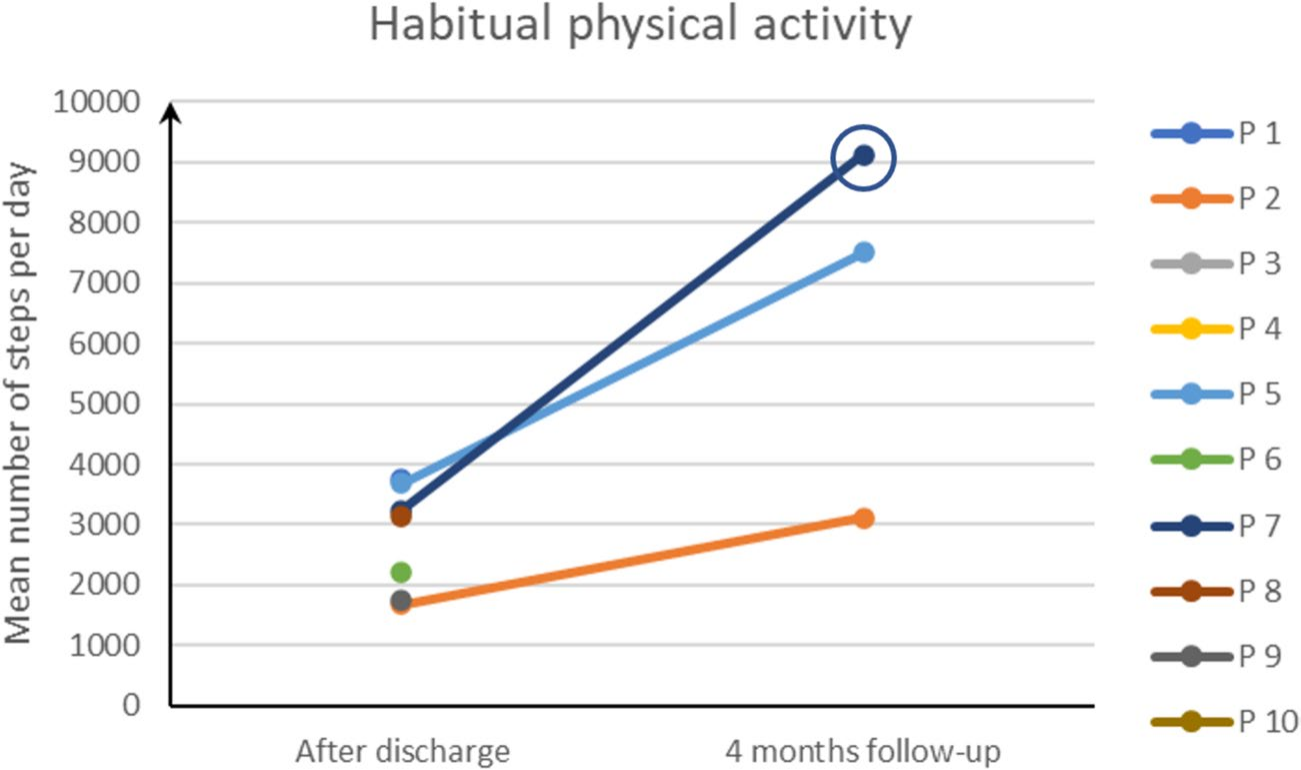
Descriptive data from activity monitor. The patient who exercised 22 out of 24 sessions has been highlighted with a circle, at 4 months follow-up

The physical tests, questionnaires, and activity monitoring showed the ability to indicate measurement changes between tests and were therefore feasible and illustrated in Figs. 2 and 3, and Table 4, respectively.

**Table 4:** Scores for the EORTC QLQ-C30 and BLM-30, presented as median (min–max)

	**Preoperative (*n* = 8)**	**At discharge (*n* = 8)**	**4-month follow-up (*n* = 4)**
**QLQ-C30**			
**Global quality of life scale**^**a**^	79 (0–92)	33 (0–83)	92 (67–100)
**Functional scales**^**a**^			
Physical functioning	87 (67–100)	43 (0–67)	90 (67–93)
Role functioning	100 (50–100)	25 (0–100)	100 (83–100)
Emotional functioning	82 (17–100)	75 (33–100)	92 (75–100)
Cognitive functioning	83 (3–100)	67 (17–100)	75 (50–100)
Social functioning	75 (0–100)	42 (0–83)	100 (67–100)
**Symptom scales**			
Fatigue	38 (0–67)	90 (23–100)	28 (0–33)
Nausea/Vomiting	0 (0–17)	33 (17–100)	0 (0–0)
Pain	9 (0–67)	75 (0–100)	0 (0–0)
Dyspnoea	17 (0–33)	50 (0–100)	33 (33–67)
Insomnia	33 (0–100)	67 (33–100)	0 (0–0)
Appetite loss	0 (0–100)	84 (33–100)	0 (0–0)
Constipation	0 (0–33)	50 (0–100)	17 (0–33)
Diarrhoea	0 (0–100)	17 (0–67)	0 (0–0)
Financial difficulties	0 (0–67)	0 (0–67)	0 (0–0)
**BLM-30**			
Urinary symptoms	58 (0–87)	0 (0–3) (n = 3)	23 (3–43) (n = 2)
Urostomy problems		10 (0–27) (n = 5)	23 (3–43) (n = 2)
Future perspective	28 (10–100)	23 (0–67)	0 (0–23)
Abdominal bloating and flatulence	9 (0–67)	67 (0–100)	0 (0–0)
Body Image	17 (0–67)	33 (10–100)	5 (0–23)
Sexual functioning, last four weeks, (n = 6)	42 (0–100)	100 (0–100)	100 (0–100)
Sexual life, last four weeks^a^	17 (0–33)	0 (0–17)	9 (0–17)

Scoring: global quality of life and functional scales range from 0 (the worst) to 100 (the best)

Symptom scales and BLM-30 scales range from 0 (the best) to 100 (the worst)

^a^From 0 (the worst) to 100 (the best)

##### Safety of the exercise programme and physical tests

No adverse events during the exercise programme were registered by PHC physiotherapists. Moreover, no adverse events were registered during the physical tests. Consequently, the exercise programme and physical tests were found to be feasible regarding safety for the patients.

##### Possibility of progression in the exercise programme

All four patients who exercised for at least 10 sessions out of the possible 24 could proceed from block one to block two within the programme, which was found to be feasible. Regarding the strengthening exercises, for 75% of the exercises that were included in the programme, the median increase was more than 50% for the patients. Aerobic interval exercise was performed by all four patients. However, the median of increased relative intensity for aerobic exercise was 0% (0–30).

## Discussion

The feasibility of an exercise intervention in PHC after RARC for UBC was supported in this study regarding safety and progression in the exercise programme. However, some of the process feasibility outcomes were hampered by the patients’ postoperative complications. Adherence to the full intervention was not feasible, mainly due to complications. Adherence to the exercise programme, the number of weeks after discharge that the patients started the programme, and the acceptability of the exercise period were all feasible but still affected by complications. Several patients were thereby prevented from fully taking part in the exercise programme.

Postoperative complications can affect several stages of an exercise intervention for this patient group. First, complications might affect adherence regarding when it is possible for the patients to begin exercise in PHC. In this study, prolonged postoperative medical care followed by care at an inpatient rehabilitation clinic affected when patients started the exercise programme. Readmission to the hospital on several occasions was also a reason for the delayed exercise start. For patients not affected by postoperative complications, it was feasible to begin exercise within the second week after discharge. This could be compared to a study where patients who had undergone RC participated in inpatient rehabilitation [38]. In that study, the patients started their rehabilitation at a median of 8 days after discharge, but 24% of the patients started after 14 days or more. However, they had a median hospital stay of 21 days, compared to patients in this study who spent 7 days, as a median, at the hospital, and some patients who spent an additional week at an inpatient rehabilitation clinic.

Second, adherence to the exercise programme and acceptability of the exercise period can be affected by medical complications. Some patients who participated in the exercise programme experienced pauses during the 12 weeks of the programme due to medical conditions but were not readmitted to the hospital. A few patients who participated in the study, where inpatient rehabilitation was evaluated, had interruptions during their rehabilitation period [38]. However, adherence to exercise is unknown and therefore also a source of potential pauses in the programme. Considering that 28% of patients were treated with antibiotics due to urinary infection, and 15% suffered from acidosis, one reason as to why they did not interrupt their rehabilitation could be that they stayed at an inpatient rehabilitation with support from health care professionals. In the model for rehabilitation after RC in a hospital setting, that we tested previously, there was a high frequency of dropouts and withdrawals due to postoperative complications [20].

Third, complications and frailty can affect the ability of patients to travel to the exercise setting. In this study, patients were required to live close to the PHC to facilitate travelling to the exercise setting. Still, patients who lived further away experienced some problems with travelling to the PHC, which affected feasibility regarding physiotherapist acceptability. In our earlier study which evaluated rehabilitation after RC, postoperative complications and impaired general condition rendered patients inability to travel twice per week to the hospital for exercise [20]. In the planned RCT, that will follow this feasibility study, patients will be able to choose the PHC that is the closest to them, from a selection of several PHCs. Living close to the rehabilitation setting has been shown to be important in increasing adherence among patients with cancer [39]. Problems regarding travel were not an issue for patients who participated in inpatient rehabilitation [38].

Due to difficulties surrounding travel, perhaps performing exercise at home is preferable for severely affected patients. In a feasibility study which evaluated a healthcare application for patients who underwent RC, 15 patients reported physical activity as the average steps and also reported vital signs before, during, and after their hospital stay [40]. After hospital discharge, 53% of patients reported their physical activity and vital signs daily. The low frequency could be explained due to the 67% of patients who experienced complications after discharge, and the 33% of patients who were readmitted to the hospital within 90 days of discharge, which is comparable to the 25% readmission rate found in this feasibility study.

Consequently, postoperative complications affect adherence to and acceptability of exercise after RARC. Complications in this patient group can also be affected by high comorbidity and mental health conditions which have been shown to worsen morbidity and mortality rates [41]. Five out of the six patients that ended their participation in this study suffered from postoperative complications. Decreased postoperative complications would likely have a positive effect on adherence. The exercise intervention, found to be feasible in this study, could theoretically have positive effects on respiration, circulation, and the immune system and thereby decrease the frequency of venous thrombosis, pulmonary complications, urinary tract infections, bowel disorders, and wound infections [7–10]. The frequencies of these complications are planned to be evaluated in the planned RCT. Future research should also address the lack of rehabilitation between hospital discharge and starting exercise at PHCs.

Some improvements will be made to the upcoming RCT as a consequence of the results of this feasibility study. Several patients ended their participation in this study, which is a known problem within exercise intervention studies for this patient group [20]. In the RCT, we plan to include more patients than that found from power calculations to account for dropouts [21]. Exclusion criteria will be improved since they were found to be infeasible and an exclusion criterion concerning cognitive impairment will be added. A patient will be excluded if the medical record shows a diagnosis, or ongoing investigation, regarding cognitive impairment. Also, as the acceptability of physical tests was not found to be feasible in this study, improvements will be made for the future RCT. Since several of the patients did not want to perform the 6-min walk test at hospital discharge due to feebleness or postoperative complications, instructions will be improved. In the instructions, it will be explained to the patients that they are not expected to walk as far as at the preoperative test. Also, a gait speed test which is assessed with the 12-m walk test, and thought to be easier for patients, will be added to provide an outcome of physical function at discharge [42].

### Strengths and limitations

One of the strengths of this feasibility study is that PHC physiotherapists were satisfied with the referrals from the hospital and perceived the main parts of the process as acceptable and thereby feasible. Another strength is that the patients paid for their visits at PHC, as they would have done in usual care. These strengths have importance for the implementation of this exercise programme in real-world standard care settings. Moreover, an objective measure of habitual physical activity was used, which overcomes the biases of self-reported physical activity [43, 44]. A limitation of this study is that it was not an RCT, which was a conscious choice. Since the main objective of this study was to evaluate the feasibility of the exercise programme, an assessment of a control group intervention was not prioritised. However, due to the known intervention, it is possible that the patients who chose to participate were highly motivated to exercise. This selection bias is a known problem with physical exercise interventions and hinders external validity, as frailer patients tend to not participate [45]. Also, patients who were invited to participate lived relatively close to the chosen PHC setting. Hence, patients were not recruited consecutively and there was no assessment of the recruitment rate, which represents limitations in this study.

## Conclusions

For patients who had undergone RARC for UBC and took part in the exercise programme, this intervention in PHC was feasible with respect to safety and progression in the exercise programme. Postoperative complications had a major effect on adherence to the intervention, which is a known problem after RARC. Outcomes that will be used for evaluations in the future RCT were all capable of indicating change between tests. Finally, this feasibility study identified several improvements to be made to the process, prior to the future RCT.

## Data Availability

The datasets generated during and/or analysed during the current study are not publicly available due to Swedish and EU personal data legislation, but are available from the corresponding author on reasonable request. Any sharing of data will be regulated via a data transfer and user agreement with the recipient.

## Abbreviations

HADS: Hospital Anxiety and Depression Scale
HRQoL: Health-related quality of life
NRS: Numeric Rating Scale
PHC: Primary health care
RARC: Robotic-assisted radical cystectomy
RC: Radical cystectomy
RCT: Randomised controlled trial
UBC: Urinary bladder cancer
